# Pyrrolizidine Alkaloid-Induced Hepatotoxicity: A Narrative Review on Molecular Mechanisms and Detoxification Strategies

**DOI:** 10.3390/antiox15050635

**Published:** 2026-05-16

**Authors:** Yizhuo Fang, Xiaosong Zhang, Chongshan Dai, Zhihui Hao

**Affiliations:** 1 Innovation Centre of Chinese Veterinary Medicine, College of Veterinary Medicine, China Agricultural University, Beijing 100193, China; 2 State Key Laboratory of Veterinary Public Health and Safety, College of Veterinary Medicine, China Agricultural University, Beijing 100193, China; 3 Key Biology Laboratory of Chinese Veterinary Medicine, Ministry of Agriculture and Rural Affairs, Beijing 100193, China

**Keywords:** pyrrolizidine alkaloids, hepatotoxicity, molecular mechanisms, oxidative stress, detoxification

## Abstract

Pyrrolizidine alkaloids (PAs), a category of naturally occurring secondary metabolites, are commonly found in various botanical sources. Accumulating evidence indicates that PAs and their biologically active metabolites can interact with cellular components and trigger a variety of toxic effects in animals and humans. Notably, PAs exhibit significant hepatotoxic potential via nutritional supplements, environmental dissemination, food chain contamination, and broader ecological pollution. In this review, we summarize PA-induced hepatotoxicity in humans and animals and the underlying molecular mechanisms. It involves oxidative stress, mitochondrial dysfunction, apoptosis, ER stress, inflammation, autophagy, and ferroptosis. Several key signaling pathways, such as nuclear factor-erythroid 2 related factor 2 (Nrf2), mitogen-activated protein kinase (MAPK), protein kinase RNA-like endoplasmic reticulum kinase (PERK), toll like receptor 4 (TLR4), nuclear factor kappa-B (NF-κB), transforming growth factor beta (TGF-β), p53, farnesoid X receptor (FXR), and pregnane X receptor (PXR), are also implicated. Furthermore, this review discusses diagnostic approaches, metabolic activation pathways, and detoxification strategies targeting PA-induced liver injury. Collectively, this review provides a comprehensive understanding of the molecular basis of PA hepatotoxicity and underscores the urgent need for improved risk assessment, early diagnosis, and effective detoxification interventions to mitigate PA-related liver diseases in humans and animals.

## 1. Introduction

**Figure 1:**
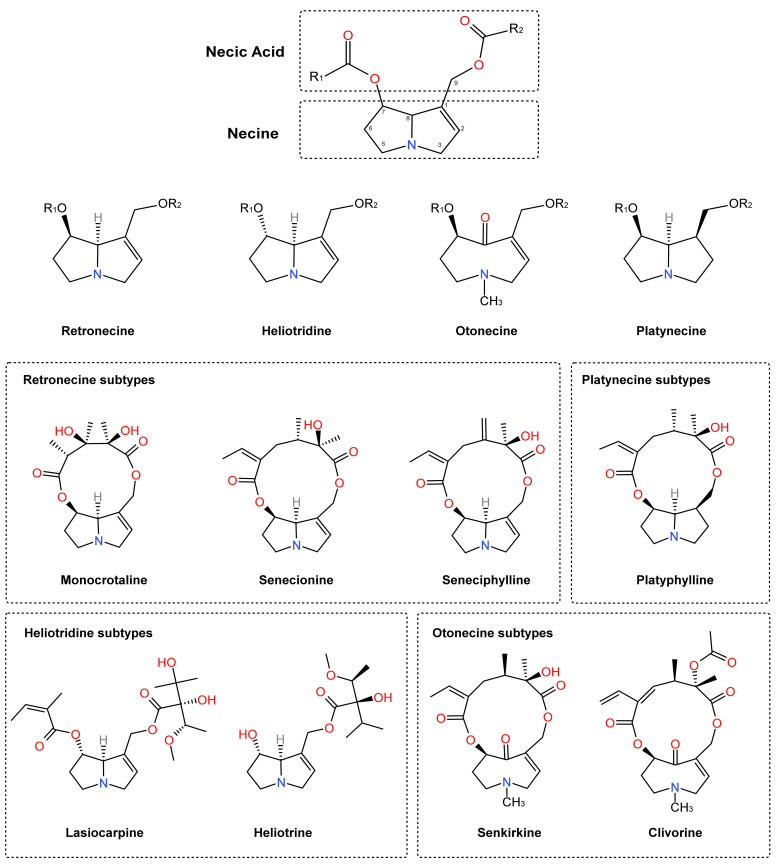
Basic structure, classification and representative members of PAs.

Pyrrolizidine alkaloids (PAs) are a class of naturally occurring plant toxins synthesized as secondary metabolites by more than 6000 plant species, predominantly within the families Asteraceae (e.g., *Senecio*), Boraginaceae (e.g., *Heliotropium*), and Fabaceae (e.g., *Crotalaria*) [1]. PAs are heterocyclic compounds that contain nitrogen, composed of necic acid and necine bases [2]. PAs can be divided further into four subtypes: retronecine, heliotridine, otonecine, and platynecine, based on variations in their necine base [2]. The first three types include C1-C2 double bonds, which are susceptible to oxidative transformation into hepatotoxic dehydropyrrolizidine (DHP) alkaloids [3]. Figure 1 shows the chemical structure, several subtypes of PAs, and the representative members of these four subtypes, including retronecine (e.g., monocrotaline, senecionine, and seneciphylline), heliotridine (e.g., lasiocarpine and heliotrine), otonecine (e.g., senkirkine and clivorine), and platynecine (e.g., platyphylline). Additionally, PA *N*-oxides, which are derivatives of PAs, are also considered the key factor in PA-related herbal-induced liver injury (HILI) [4,5].

The rapid globalization of agricultural trade and increasing consumption of plant-based products, along with the potent hepatotoxic and carcinogenic properties, have heightened attention on the toxicology and risk assessment of PAs. Humans are exposed to PAs by the direct ingestion of herbs, teas, dietary supplements, and other food matrices containing PAs. Indirect exposure may also occur through the consumption of contaminated animal-derived products such as meat, honey, and milk [6,7,8,9]. Moreover, PA residues from plant materials can translocate into the tissues of neighboring plants via soil-mediated transfer [8]. High levels of PAs (up to 123.3 μg/kg) have been detected in dried tea across multiple regions in China [7]. Additionally, chronic and repeated exposure to PAs can increase the half-life of toxic metabolites and cause long-term risk to both humans and animals [10].

Since the 1960s, the toxicological profile of PAs, encompassing hepatotoxicity, pulmonary toxicity, and genotoxicity, has been preliminarily validated [11,12,13]. Epidemiological investigations indicate that exposure to PAs might induce hepatic sinusoidal obstruction syndrome (HSOS) in humans, which is characterized by abnormal clinical signs such as abdominal distension, hepatic pain, ascites, jaundice, and hepatomegaly [14,15,16]. Previous studies suggest that the liver is a principal target organ for PA-induced toxic effects [17,18]. Its bioactivation process is mainly mediated by the cytochrome P450 enzymes (CYPs), and this process can convert prototypical PAs into DHP, an electrophilic metabolite. DHP has been observed to covalently bind to nucleic acids, proteins, and other biomolecules, which ultimately causes toxic effects [17,18]. To date, the exact molecular mechanisms of HILI caused by PA exposure remain unclear.

In this review, we summarize PA-induced hepatotoxicity in humans and animals and the underlying molecular mechanisms. We also discuss diagnostic approaches, metabolic activation pathways, and detoxification strategies targeting PA-induced liver injury. We hope this review can provide a comprehensive understanding of the molecular basis of PA hepatotoxicity and underscore the urgent need for improved risk assessment, early diagnosis, and effective detoxification interventions to mitigate PA-related liver diseases in humans and animals.

## 2. Overview of PA-Induced Hepatotoxic Effects and Diagnosis

PAs exert certain toxic effects on insects, birds, mammals, and other organisms [19,20,21]. Comprehensive studies indicate that exposure to PAs can induce liver damage accompanied by several typical clinical signs, such as abdominal distension, ascites, and jaundice as well as biochemical abnormalities (e.g., aspartate transaminase (AST), alanine transaminase (ALT), alkaline phosphatase (ALP), gamma-glutamyl transferase (GGT), lactate dehydrogenase (LDH), albumin (ALB), direct bilirubin (DB), total bilirubin (TBIL), total bile acid (TBA), and total protein (TP)), which reflect impaired liver function. Exposure to PAs has been documented to induce various histopathological lesions, including necrosis of sinusoidal endothelial cells and hepatocytes, sinusoidal dilatation, hemorrhage, occasional inflammatory cell infiltration, and fibrosis in the liver tissue of mice or rats [22,23]. In addition, PA-related HILI is dependent on several key factors, including exposure duration, dosage, gender, and species susceptibility [21,24,25,26,27]. Table 1 presents a systematic summary documenting clinical and experimental data from PA-induced HILI in humans and animals (such as cattle, horses, rats, and mice).

The Roussel Uclaf Causality Assessment Method (RUCAM), developed in the 1990s, is a rigorous and structured quantitative causality assessment tool, utilized to differentiate drug-induced liver injury and HILI from viral hepatitis or other cases of liver injury [52,53]. After decades of clinical validation, it has established itself as the gold standard for DILI and HILI diagnosis, with a significant upgrade in 2016 [52].

Moreover, the HSOS caused by PAs markedly varies from that induced by hematopoietic stem cell transplantation in terms of epidemiology, ethnicity, etiology, and underlying illnesses. In Nanjing, the Committee of Hepatobiliary Diseases of the Chinese Society of Gastroenterology proposed a qualitative diagnostic criterion for PAs-HSOS grounded in the evidence-based medical data in 2017 [15]. A retrospective investigation verified the clinical diagnostic accuracy of the Nanjing criteria, demonstrating a high efficacy in detecting PAs-HSOS, with a sensitivity of 95.35% and a specificity of 100% [23]. Nonetheless, one significant limitation is that the Nanjing criteria currently lack the requisite causality assessment method; integrating such a module would surely improve diagnostic accuracy.

It is crucial to note that the challenges in tracking illness progression, equipment conditions, and physiological variations render the RUCAM and other causality assessment methods inapplicable to PA-related HILI in animals, particularly in grazing animals. These limitations, in turn, adversely affect the quality and reliability of the published data.

## 3. Metabolism and Biotransformation of PAs

### 3.1. Metabolism and Biological Activation of PAs

Studies indicate that the metabolism of PAs in mammals involves various pathways. Carboxylesterases and hydrolases catalyze the hydrolysis of PAs, through which PAs are cleaved into necine bases and necic acids. Studies on specific enzyme inhibitors have validated this metabolic process. Variations in esterase activity across different species are associated with resistance to hepatotoxicity induced by PAs [54,55,56]. For example, guinea pigs exhibit tolerance to PA-induced hepatotoxicity, which is attributed to the expression of a specific, highly active carboxylesterase isoform named GPH1 that accelerates the metabolic conversion and detoxification of PAs [55,57].

The *N*-oxidation of PAs, catalyzed by cytochrome P450 (CYP) or flavin-containing monooxygenases (FMO), is considered another metabolic detoxification process for PAs [58,59]. Conversely, the reduction of PA *N*-oxides reverts them to parent PAs and constitutes a major step associated with the activation of hepatotoxicity [25]. PA *N*-oxides are rapidly converted to the corresponding parent PAs within a few hours in rat liver. This process is mediated by nicotinamide adenine dinucleotide phosphate (NADPH)-dependent CYP1A2 and CYP2D6 metabolic enzymes, along with gut microbiota [60]. Meanwhile, the tissue oxygen partial pressure significantly influences the balance between the *N*-oxidation and reduction of the necine in PAs [60].

The formation of conjugated double bonds, subsequent to the oxidation reaction of PAs, is regarded as the key process in the bioactivation of PAs [61]. The activated product DHP is generated from the parent PAs via CYP catalysis. DHP has highly reactive electrophilic properties, enabling it to readily form covalent adducts with DNA, RNA, proteins, and other biomolecules. This leads to various adverse effects in PA-related HILI [62,63]. CYP-knockout murine models further validate this mechanism [64,65]. Nevertheless, PA hepatotoxicity can occur independently of dehydrogenation, which is potentially attributed to the direct targeted effects or unknown mechanisms. Likewise, some PAs (such as clivorine and irniine) directly cause cytotoxicity in cell lines, such as HEK293, HeLa, and RLEC, which lack hepatic metabolic enzyme activity [66,67].

**Figure 2:**
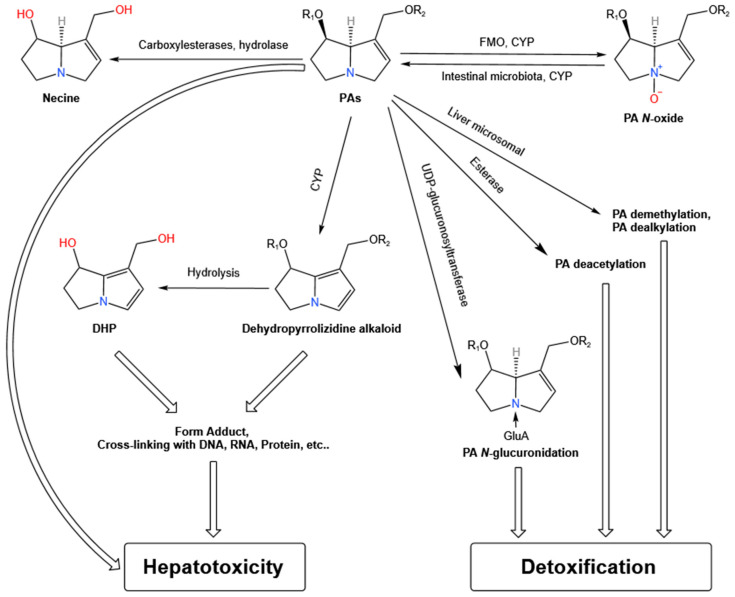
The major metabolism and biotransformation pathways of PAs. GluA, glucuronic acid; FMO, flavin-containing monooxygenase; CYP, cytochrome P450 enzymes; PAs, pyrrolizidine alkaloids; DHP, Dehydropyrrolizidine.

Other hepatic metabolic pathways of PAs include *N*-glucuronidation, deacetylation, demethylation, and dealkylation. *N*-glucuronidation is particularly prominent in humans, rabbits, cattle, sheep, and pigs. The activity of this pathway correlates with interspecies differences in susceptibility to PA-induced hepatotoxicity [68]. *N*-glucuronidation is mediated by UDP-glucuronosyltransferase 1A4 in humans. Transgenic mice expressing UDP-glucuronosyltransferase 1A4 exhibit resistance to senecionine, in contrast to the wild type, and this illustrates the detoxifying role of *N*-glucuronidation [69]. Results from in vivo and in vitro studies indicate that the acetyl group on the macrocyclic ester of PAs significantly contributes to their hepatotoxicity [70]. Deacetylation and demethylation occur on the acetyl substituent of the necic acid moiety, and the studies on the transformation of open-chain diester PAs in hepatic microsomes have shown that demethylation and dealkylation reactions result in shortened necic acids [71]. Although these reactions are not dominant routes, they collectively contribute to the detoxification process. Figure 2 shows the general metabolism and biotransformation of PAs.

### 3.2. Binding Reactions in Necine Bases of PAs

#### 3.2.1. Formation of Adducts Between PAs and DNA, Proteins, and Other Biomolecules

DHP displays electrophilic activity at its C9 and C7 carbon positions, enabling the rapid formation of diverse adducts with nucleophiles, such as thiols, amines, and hydroxyls [72]. PAs adducts, formed upon interaction with DNA nucleotides, are considered biomarkers of PA-induced DNA damage [73]. Riddelliine forms adducts with deoxyadenosine and deoxyguanosine in the presence of rat liver microsomes and calf thymus DNA, exhibiting preferential binding at the C9 site [74]. This preferential reactivity may be attributed to steric hindrance effects and the conformational characteristics of the DNA bases [74].

DHP covalently binds to proteins and forms protein adducts. Protein adducts result in dysfunction of the targeted protein or disruption of its expression [75]. Dehydrosenecionine has been demonstrated to bind to thrombospondin 1 (TSP1), a type of matricellular glycoprotein mediating interactions between cells and the matrix, and then upregulates the expression of TSP1 protein and induces severe liver injury in mice [76]. Based on proteomic differential analysis, Lin et al. reveal that retrorsine can directly interact with ATP synthase subunit β, and it triggers mitochondrial-mediated apoptotic cell death in rat liver [77,78]. The level of protein adducts is recognized as a reliable PA-specific biomarker in several studies [14,79,80,81].

Recent studies have confirmed the hypothesis that DHP can bind to RNA and exhibits the strongest binding affinity towards guanine nucleotides [82]. RNA adducts suppress methylation levels and reverse transcription characteristics. Such alterations lead to mRNA translation arrest, RNA polymerization, and resistance to ribonucleases [82]. Although the biochemical consequences of RNA adduction are well characterized, their subsequent effects on the organisms and metabolic fate remain to be elucidated.

DHP forms conjugates with glutathione (GSH), several amino acids, or their derivatives [83,84,85,86]. Supplementation with GSH or cysteine competitively reduces DNA adduct formation in primary cells [86]. The resultant products exhibit lower hepatotoxicity and imply a potential detoxification pathway [86]. Nevertheless, the same research team shows that 7-GSH-DHP and 7-cysteine-DHP retain DNA adduct-forming capacity and still exert residual hepatotoxicity, which suggests a hepatotoxicity risk under prolonged exposure even after GSH intervention [87].

#### 3.2.2. PA-Induced Macromolecular Cross-Linking

PAs inherently possess the potential for bimolecular cross-linking, due to the capability to generate adducts at the C9 and C7 positions. Experimental evidence exists about the formation of DNA-DNA and DNA–protein cross-linking complexes [88,89,90,91]. Cross-linking activity is closely associated with disrupted mitotic recombination, cell cycle arrest, somatic cell mutations, and cytotoxicity [92,93]. Administration of dehydrosenecionine or dehydromonocrotaline at high dose ratios (1:1 and 1:2; DNA/DHP, *w*/*w*) for 2 h induces DNA-DNA cross-links and completely terminates DNA amplification, which indicates their antimitotic activity [94]. In CYP3A4-overexpressing HepG2 cells, Ingenuity Pathway Analysis (IPA) reveals that lasiocarpine or riddelliine treatment for 24 h dysregulates the expression of genes linked to DNA damage repair and cell cycle regulation. This includes an increase in *CDKN1A* and *GADD45A*, and a decrease in *PLK1*, *CCNB1*, *CCNB2*, *CDK1*, *CHEK1*, and *CDK2*, in a dose-dependent manner [95]. The chromosomal defects observed under microscopy in the same study foreshadow adverse outcomes following DNA cross-linking induced by PAs [95].

Differences in nucleophilic strength among protein residues critically influence the formation of PA–protein cross-links. Dehydromonocrotaline shows no interaction preference with various DNA sequences [96]. However, certain nucleophiles (e.g., cysteine, aspartic acid, and GSH) engage competitively in cross-linking with DNA, which correlates with structural nucleophilic activity [96]. Additionally, the potential for cross-linking between DNA and protein in Kim’s investigation is consistent with known ranks of PA-related HILI effect in animal models [91].

## 4. Molecular Mechanisms of PA-Induced Hepatotoxicity

### 4.1. Oxidative Stress

Oxidative stress, a disruption in redox homeostasis, contributes to multiple forms of liver injury [97]. Reactive oxygen species (ROS) are highly reactive oxygen-derived molecules originating from the incomplete reduction of molecular oxygen (e.g., H_2_O_2_ and hydroxyl radicals). ROS mediate cellular signaling, transcriptional regulation, and immune modulation at physiological levels, yet their overproduction provokes hepatic oxidative stress. Accumulating evidence indicates that ROS overproduction occurs following PA exposure in hepatocyte-derived cell lines and primary rat hepatocytes [98,99].

Aerobic organisms possess intricate antioxidant defense systems, including glutathione peroxidase (GPx), glutathione S-transferase (GST), glutathione reductase (GR), catalase (CAT), and superoxide dismutase (SOD), that counteract ROS-mediated cellular oxidation. Excessive ROS and free radicals attack intracellular lipids and propagate oxidative damage through lipid peroxidation chain reactions, which generate malondialdehyde (MDA, primary biomarker of lipid peroxidation) [100]. Significant increases in MDA levels, suppression of GSH-associated enzyme activities (such as GPx, GST, and GR), and abnormal changes in antioxidant enzyme activities (CAT, SOD) are observed in rat primary hepatocytes treated with clivorine at doses of 1–100 μM for 24 h [101]. Comparable alterations of tissue total antioxidant capacity, MDA, GPx, GST, and CAT are evident in mice administered oral isoline at 100 mg/kg for 36 h [102]. Wang’s team demonstrates that oral administration of isoline at 110 mg/kg for 36 h upregulates GST-π and GPx1 protein levels in the liver of mice [103]. The in silico method further reveals complex binding modes between these proteins and DHP [103,104]. This molecular interaction supports GSH-mediated conjugation and detoxification, which contributes to cellular defense against PA-induced oxidative stress.

The mechanism of inhibiting SOD activity is related to the sirtuin (SIRT) 3-SOD2-mitochondrial ROS pathway. Specifically, PAs downregulate the expression of SIRT3, blocking the deacetylation of SOD2 [105]. This further suppresses the elimination of mitochondrial ROS and ultimately leads to apoptosis [105]. The regulation of SIRT3 in the upstream likely involves interaction with Forkhead Box Protein O (FoxO) 3a, an oxidative stress-responsive regulator. SIRT3-dependent deacetylation of FoxO3a modulates its nuclear translocation and downregulates transcription of antioxidant genes such as SOD and CAT [106]. Additionally, SIRT3 directly affects mitochondrial ROS generation by modulating protein acetylation along the electron transport chain [106]. Conversely, PAs target another key transcription factor, nuclear factor erythroid 2-related factor 2 (Nrf2). The intracellular abundance of Nrf2 is negatively regulated by Kelch-like ECH-associated protein 1 (Keap1) physiologically. In response to oxidative stress, the complex of Keap1 and Nrf2 is dismantled, and this promotes Nrf2 nuclear translocation. Nrf2 dimerizes with small MAF proteins and binds to antioxidant response elements (AREs), which mediates a series of processes including detoxification, antioxidant responses, NADPH regeneration, and xenobiotic metabolism [107]. Administration of monocrotaline at 360 mg/kg for 24 h induces elevation of AST and ALT levels and loss of hepatic sinusoidal fenestration in Nrf2-knockout mice [108]. Moreover, the decrease in mRNA expression of Nrf2, glutamate–cysteine ligase catalytic subunit (GLCC), and glutamate–cysteine ligase modifier subunit (GLCM) is observed in the liver tissues of rats orally administered monocrotaline (at 90 mg/kg for 48 h) or retrorsine (at 40 mg/kg for 48 h) [109,110]. Similarly, monocrotaline treatment at 90 mg/kg for 48 h significantly blocks Nrf2 nuclear translocation and ARE-responsive gene (e.g., Nrf2, GCLC, GCLM, and NQO-1) expression in the livers of rats [111]. Taken together, these findings indicate that PA exposure may interfere with the SIRT3/FoxO3a and Nrf2-ARE systems and induce oxidative stress, finally triggering hepatotoxicity.

Complex disruption of the glutathione (GSH) antioxidant system deeply contributes to PA hepatotoxicity. GSH, the most crucial antioxidant, can bind to toxic substances, ROS, and other agents that induce oxidative stress [112]. Research has shown that exposure to PAs depletes hepatic GSH in a dose- and time-dependent manner [113,114,115,116]. Adonifoline, senecionine, or monocrotaline (25–100 μM for 72 h) lowers GSH/Glutathione oxidized (GSSG) ratios in L-02 hepatocytes. The inhibitory effect and cytotoxicity are enhanced by buthionine sulfoximine (BSO, an irreversible inhibitor of intracellular GSH synthesis), whereas supplementation with GSH or its precursor N-acetylcysteine (NAC) reverses these effects [114]. Treatment with isoline (at 110 mg/kg for 8 h) suppresses the glutamate–cysteine ligase (GCL, the rate-limiting enzyme for GSH biosynthesis) activity and its expression in mice [24].

**Figure 3:**
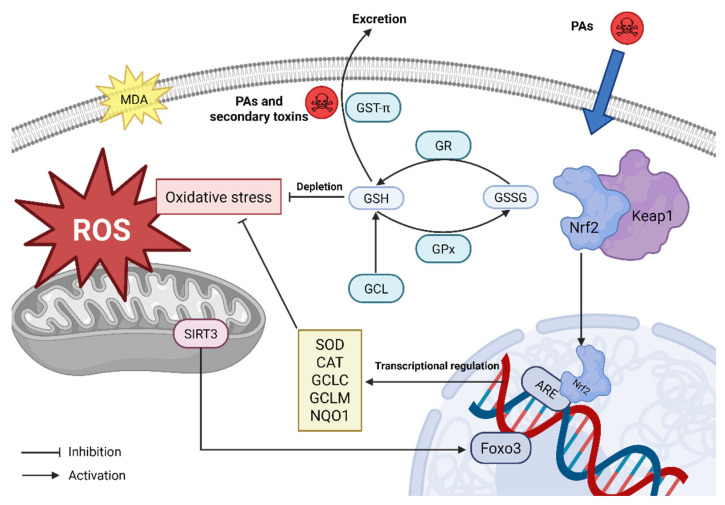
Molecular mechanism of oxidative stress in PA hepatotoxicity. ROS, reactive oxygen species; PAs, pyrrolizidine alkaloids; MDA, malondialdehyde; SIRT3, Sirtuin 3; FoxO3, forkhead box protein O3; GSH, glutathione; GCL, glutathione–cysteine lyase; GPx, glutathione peroxidase; GSSG, glutathione oxidized; GR, gluathione reductase; GST-π, glutathione S-transferase π; Keap1, Kelch-like ECH-associated protein 1; Nrf2, nuclear factor erythroid 2-related factor 2; ARE, antioxidant response element; SOD, superoxide dismutase; CAT, catalase; GCLC, glutamate–cysteine ligase catalytic subunit; GCLM, glutamate–cysteine ligase modifier subunit; NQO1, NAD(P)H dehydrogenase quinone 1.

In summary, PA-related HILI is mediated by the production of ROS, GSH depletion, antioxidant enzyme inhibition, and dysfunction of the Keap1/Nrf2 and SIRT3/FoxO3a systems (Figure 3).

### 4.2. Mitochondrial Dysfunction and Apoptosis

Research has demonstrated that PAs or PA-containing plants induce apoptosis in the liver, as verified by histopathology, immunostaining, and specific inhibitors [117,118,119]. In general, PAs trigger apoptosis via both the extrinsic and intrinsic pathways.

Intrinsic apoptosis is initiated by polymerization of the multidomain pro-apoptotic molecules Bcl2-associated X (Bax) and Bcl-2 homologous antagonist killer (Bak). The ratio of these proteins to their corresponding anti-apoptotic counterparts, B-cell lymphoma-extra-large (Bcl-XL) or B-cell lymphoma-2 (Bcl-2), largely determines the final outcome in the apoptotic process [120]. Relocalization of Bax from the cytoplasm to mitochondria further induces mitochondrial dysfunction and release of Cytc. Cytosolic Cytc assembles into apoptosomes with apoptotic protease activating factor-1 and pro-caspase-9, ultimately activating the executioner caspase-3 [120]. Retrorsine exposure at 30 mg/kg via intraperitoneal injection for 14 days significantly elevated the Bax/Bcl-XL protein ratio, Bax mitochondrial translocation, and Cytc leakage and finally caused hepatocyte apoptosis in rats [121]. Monocrotaline treatment at 50–200 mg/kg dose-dependently increased TUNEL-positive nuclei and Bax/Bcl-2 immunostaining in rat livers after 18th hours and 6 weeks at the end of the experiment [122]. Treatment of clivorine at 1–100 μM for 48 h did not suppress Bcl-XL mRNA, but rather accelerated its ubiquitin-dependent proteasomal degradation, and augmented caspase-3/-9 cleavage and mitochondrial Cytc release in L-02 cells [123]. Furthermore, clivorine treatment of 100 μM transiently suppresses tumor protein 53 (p53, a pivotal regulator of the Bcl-XL anti-apoptotic pathway) within 3–6 h, and it potentially reflects an early anti-apoptotic adaptation [124]. This research excluded the influence of clivorine on Bcl-2 expression [124]. Meanwhile, treatment of lasiocarpine (5 μM for 24 h) or heliotrine (50 μM for 24 h) raised p53 protein levels in CYP3A4-overexpressing HepG2 cells [125]. Following 4 weeks of exposure to five PAs (echimidine, heliotrine, lasiocarpine, senecionine, and senkirkine) at 3.3 mg/kg, RNA microarray analysis and IPA revealed the activation of the p53 signaling pathway in rats, with the highest z-score [126]. Mechanistically, PA derivatives selectively bind to mouse double minute 2 homolog (MDM2, a negative regulator of p53), and lead to the downregulation of MDM2, activation of the MDM2/p53 axis, and induction of wild-type p53-dependent apoptosis [127].

Altered mitochondrial permeability is an irreversible step in intrinsic apoptosis [128]. Exposure to PAs resulted in alterations in mitochondrial membrane potential in HepaRG and HepD cells [98,129]. Retrorsine treatment at 120 μM for 6 h depolarizes mitochondrial transmembrane and halts mitochondrial respiration in HepaRG cells, while excluding the interference of non-mitochondrial respiration or glycolytic suppression by monitoring of extracellular acidification [77]. The rescue effect of galactose culture medium or mitochondrial permeability inhibitor olesoxime further demonstrated that mitochondria serve as the prime PA-targeted organelle [77]. PAs also regulate dynamin-related protein 1 (Drp1), which can drive pathological mitochondrial fission and cristae remodeling, crucial for mitochondrial outer membrane permeabilization [130]. Treatment with seneciphylline or senecionine at 20 μM for 24 h regulates mitochondrial translocation, and it induces membrane potential disruption, mitochondrial fragmentation, and apoptosis in primary mouse hepatocytes [131,132]. The cotreatment with a Drp1 inhibitor, Mdivi-1, reversed the above impairments, which indicates that mitochondrial dynamics play a pivotal role in cytotoxicity caused by PAs exposure [131,132].

Extrinsic apoptosis begins with the activation of cell surface death receptors, the assembly of death-inducing signaling complexes, and subsequent activation of caspase-8. Echimidine and senecionine treatment at 5, 35, or 70 μM for 6 h dose-dependently elevates caspase-8 activity and mRNA expression in HepaRG cells [129]. As signaling molecules in the extrinsic apoptosis pathway, both Fas-associated death domain (FADD) and TNFRSF1A-associated death domain (TRADD) participate in distinct death receptor pathways [120]. In the Fas-mediated pathway, the Fas cell surface death receptor (Fas) on the plasma membrane directly interacts with the FADD, resulting in caspase-8 activation and initiation of apoptosis. In the tumor necrosis factor (TNF) receptor 1-mediated pathway, tumor necrosis factor receptors recruit TRADD as an adaptor and indirectly link FADD to regulate caspase-8 activity. Simultaneously, it has been demonstrated that TRADD can directly bind to TNF receptor-associated factor 2 (TRAF2) or the death domain kinase receptor-interacting protein (RIP), both of which are essential for activating the cascading branches of mitogen-activated protein kinase (MAPK) [133]. Gluck et al. revealed that the treatment of different structural PAs disrupts mRNA levels of extrinsic apoptosis-related factors, such as FADD, TRADD, and TRAF2 [134]. For example, Ebmeyer et al. found that treatment of lasiocarpine at 0–200 μM for 24 h significantly elevates the expression of Fas and caspase-8 mRNAs and proteins in HepG2 cells [135]. Intraperitoneal injection of monocrotaline at 270 mg/kg for 48 h increases both Fas and platelet Fas ligand (FasL) protein expression in the livers of mice [136]. A novel pathogenic mechanism underlying HSOS involves platelet-derived FasL interacting with hepatocyte Fas, which promotes extrinsic apoptotic signaling. This mechanism may explain the lack of therapeutic efficacy observed with treatment using the caspase-8 inhibitor Ac-IETD-pNA (20 μM for 50 h) [123,136].

As an evolutionarily conserved family of serine/threonine/tyrosine-phosphorylating enzymes, MAPKs regulate diverse processes, including apoptosis [100]. All three MAPK branch cascades, extracellular signal-regulated kinase (ERK), c-Jun N-terminal kinase (JNK), and mitogen-activated protein kinase 14 (i.e., p38 MAPK), are activated in PA-related HILI. Clivorine exposure at 100 μM for 3–60 min significantly increases the phosphorylation level of p38 in L-02 cells [66], and this cytotoxicity is independent of CYP-mediated activation [137]. Pyrrolizidine derivative treatment at 25 μM for 24 h induces the phosphorylation of upstream mitogen-activated protein kinase kinase 4 and mitogen-activated protein kinase kinase 7, which sustains activation of JNK1/2 and downstream target protein c-Jun, and leads to cell cycle arrest and apoptosis [127]. The pretreatment with the respective inhibitors FR180204 (ERK inhibitor), SP600125 (JNK inhibitor), or SB202190 (p38 inhibitor) reverses retrorsine-induced apoptosis within 1 h [99]. Theories suggest that during intrinsic apoptosis, JNK and p38 regulate transcription factor activator protein-1 or p53 phosphorylation. The dimerization of activated p53 and p73 facilitates the expression of pro-apoptotic genes *puma* and *Bax* [138]. Furthermore, evidence indicates that JNK and p38 directly promote the translocation of Bax to mitochondria [139,140]. During extrinsic apoptosis, JNK and p38 promote the ubiquitin-mediated degradation of cellular FLICE-like inhibitory protein, then activate apoptosis [141]. Pharmacological inhibition of JNK markedly attenuates seneciphylline-induced mitochondrial depolarization and apoptosis in primary mouse hepatocytes [131]. Although key nodes of the aforementioned mechanisms participate in PA-related HILI, their interrelationships require further validation. Zhu et al. identify ROS as the initiator, as to the interpretations of activation in MAPK upstream in retrorsine-induced HILI [99]. During this ROS/MAPK cascade, ERK, JNK, and p38 are phosphorylated and activated in primary rat hepatocytes [99]. Undoubtedly, the introduction of MAPK broadens the understanding of the mechanisms underlying apoptosis in PA-related HILI.

**Figure 4:**
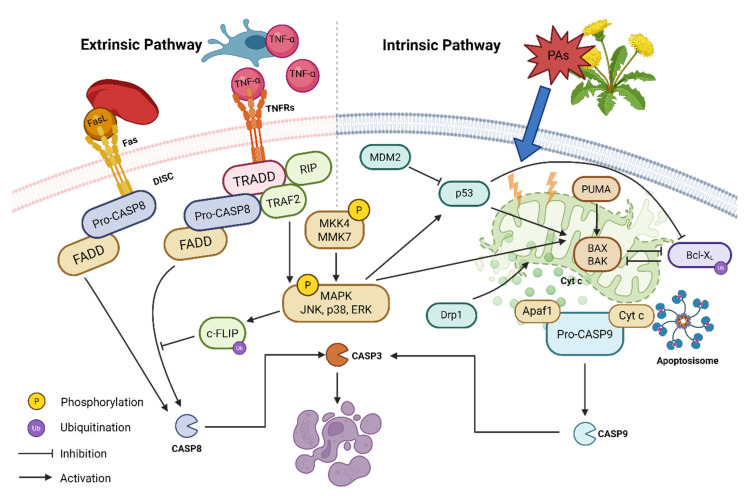
Molecular mechanisms of mitochondrial dysfunction and apoptosis in PAs hepatotoxicity including extrinsic and intrinsic pathways. PAs, pyrrolizidine alkaloids; Fas, factor-related apoptosis; FasL, factor-related apoptosis ligand; DISC, death-inducing signaling complex; CASP8, caspase-8; FADD, fas-associated death domain; TRADD, TNFRSF1A-associated death domain; TNF-α, tumor necrosis factor-α; TNFRs, tumor necrosis factor receptors; RIP, receptor-interacting protein; TRAF2, TNF receptor-associated factor 2; c-FLIP, cellular FLICE-like inhibitory protein; MAPK, mitogen-activated protein kinase; JNK, c-Jun N-terminal kinase; p38, mitogen-activated protein kinase 14; ERK, extracellular signal-regulated kinase; MKK4, mitogen-activated protein kinase kinase 4; MKK7, mitogen-activated protein kinase kinase 7; MDM2, mouse double minute 2 homolog; p53, tumor protein 53; Drp1, dynamin-related protein 1; Bax, BCL2-associated X; BAK, Bcl-2 homologous antagonist killer; PUMA, p53 upregulated modulator of apoptosis; Apaf1, apoptotic protease activating factor 1; Cytc, cytochrome c; Bcl-XL, B-cell lymphoma-extra-large; CASP3, caspase-3.

In brief, PAs trigger apoptosis through both intrinsic and extrinsic pathways. The extrinsic apoptosis pathway involves the activation of Fas and TNF receptor cascades. The intrinsic apoptosis primarily entails mitochondrial dysfunction and permeability alteration, the imbalance of the ratio of the pro-apoptotic and anti-apoptotic proteins, and the regulation of Drp1, p53 signaling pathway, and the MAPK family (Figure 4).

### 4.3. ER Stress

ER is a cellular factory for protein synthesis, folding, modification, and processing, while being vulnerable to oxidative stress, calcium homeostasis disruption, and protein folding defects [142]. In PA-related HILI, ER stress is frequently followed by apoptotic outcomes [143]. Treatment of intermedine and lycopsamine mixture at 20–100 μg/mL for 24 h depletes Ca^2+^ stores and induces ER–mitochondrial structural cross-linking in HepD cells, which is recognized as an early sign of mitochondrial apoptosis [144]. Oral administration of monocrotaline at 45–90 mg/kg for 48 h dose-dependently accumulates the ER stress chaperone glucose-regulated protein 78 and stress sensors, including protein kinase R-like ER kinase (PERK), activating transcription factor (ATF) 6, and inositol-requiring enzyme 1 (IRE1) α in rat livers [145], and the canonical ER stress-mediated apoptotic cascade PERK/eukaryotic initiation factor 2α (eIF2α)/ATF 4/C/EBP homologous protein (CHOP) is significantly activated [145]. Sustained ER stress drives PERK homodimerization and phosphorylation. It phosphorylates eIF2α, enhances ATF4 activity, and subsequently transcriptionally upregulates CHOP [146]. CHOP orchestrates multiple protein kinases as an intermediary factor in ER stress-mediated responses such as apoptosis and autophagy [147]. Evidence indicates that siRNA-mediated suppression of CHOP reverses monocrotaline-induced apoptosis in primary rat hepatocytes [148]. Moreover, oral administration of monocrotaline at 45–90 mg/kg for 48 h upregulates the expression of caspase-12, another key molecule that controls ER stress-mediated apoptosis, in rat liver [145,149,150]. Zhang et al. find that monocrotaline treatment at 800 mg/kg via oral administration for 24 h significantly promotes flavin-containing monooxygenases 3 (FMO3) to bind cAMP-response element binding protein 3 (CREB3). It blocks nuclear translation of CREB3 and reduces transcription of prolyl 4-hydroxylase subunit β, an enzyme critical for correct protein folding, and initiates ER stress [151].

**Figure 5:**
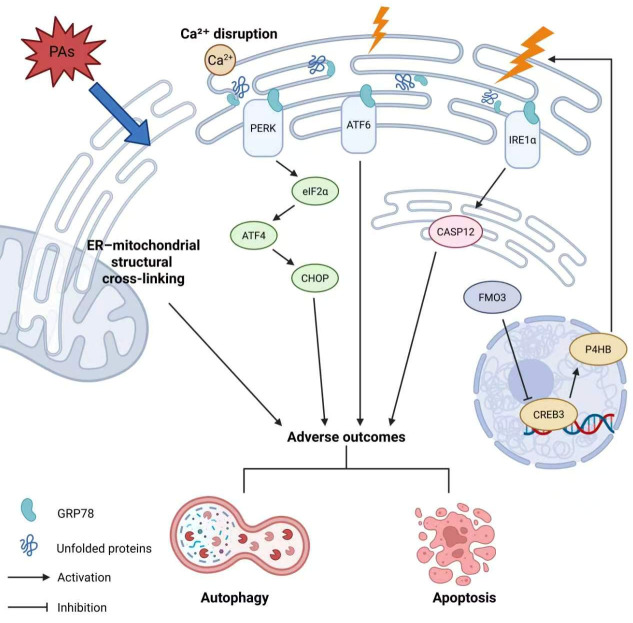
Molecular mechanisms of ER stress in PA-induced hepatotoxicity. PAs, pyrrolizidine alkaloids; PERK, protein kinase R-like ER kinase; ATF6, activating transcription factor 6; IRE1α, inositol-requiring enzyme 1 α; CASP12, Caspase-12; ATF4, activating transcription factor 4; CHOP, C/EBP homologous protein; FMO3, flavin-containing monooxygenases 3; CREB3, cAMP-response element binding protein 3; P4HB, prolyl 4-hydroxylase subunit β; GRP78, glucose-regulated protein 78.

In summary, the comprehensive activation of ER stress in PA-induced hepatotoxicity encompasses all three branches (i.e., PERK, ATF6, and IRE1α). It requires further validation of detailed activation branches other than the PERK/eIF2α/ATF4/CHOP cascade. Additionally, FMO3-mediated regulation of the CREB3 offers a broader perspective on ER stress triggering (Figure 5).

### 4.4. Inflammation and Fibrosis

Infiltration of inflammatory cells and upregulation of pro-inflammatory cytokines are commonly associated with PA hepatotoxicity. Damage-associated molecular patterns (DAMPs) can be produced by necrotic or severely damaged cells. After recognition of DAMPs, Toll-like receptor 4 (TLR4) drives myeloid differentiation primary response 88 (MyD88) or TIR-domain-containing adaptor-inducing interferon-β (TRIF) pathways [108]. It ultimately activates downstream nuclear factor kappa-B (NF-κB) and transcription of diverse pro-inflammatory cytokines, chemokines, and adhesion molecules [152]. Research aligns these PA-induced sterile inflammation with DAMPs-TLR4-MyD88(TRIF)-NF-κB signaling cascade. According to Huang et al., oral administration of monocrotaline at 300–400 mg/kg for 48 h significantly induces MyD88- and TRIF-dependent HSOS in the livers of mice [108]. Additionally, Nrf2 knockout mice exhibit elevated DAMPs (e.g., HSP60 and HMGB1) and HSOS symptoms. This implies that Nrf2 may regulate the release of DAMP molecules in the monocrotaline-induced HILI [108]. Due to the metabolic reabsorption of PAs, intestinal barrier damage, and the sensitization from intestinal inflammation, the gut–liver axis may further amplify systemic inflammation and liver injury. Oral retrorsine administration at 100 mg/kg for 24 h can induce microbiota dysbiosis, with markedly increased abundances of Bacteroides and Shigella in mice [153]. These Gram-negative bacteria elevate serum LPS levels via the gut–liver axis and activate the hepatic NF-κB pathway [153]. NF-κB-activated Kupffer cells then release TNF-α and promote hepatic inflammation and hepatocyte death [154].

Fibrogenic changes, including collagen deposition, are frequently reported in animal models of PA toxicity. These alterations are often accompanied by elevated serum fibrosis-associated cytokine levels (e.g., intercellular adhesion molecule-1 and P-selectin) and equilibrium collapse between matrix metalloproteinases and tissue inhibitors of metalloproteinases [38,39,40,42,155,156,157]. During a 14-week chronic experiment, retrorsine exposure at 40 mg/kg can induce fibrosis in the liver tissues of rats, and this process involves the activation of the canonical transforming growth factor-β (TGF-β)/mothers against decapentaplegic homolog 3 (Smad3) pathway [157]. TGF-β from platelets and myofibroblasts participates in intracellular signaling via Smad phosphorylation and regulates the expression of downstream genes, particularly collagen [157]. Canugovi et al. demonstrate that *Gynura rhizoma* extract (oral administration, at 1.0 g/kg/d for 40 successive days) develops HSOS-like symptoms in mice, with increased hepatic hydroxyproline, mRNA levels of collagen I/III, and α-smooth muscle actin immunoreactivity (fibrosis biomarker) [42]. Of note, the TGF-β/Smad3 pathway drives upregulation of TNF-α, interleukin-1β, and interleukin-6 mRNA levels [42]. Moreover, TGF-β1 also mediates fibrosis through the phosphoinositide 3-kinase (PI3K)/protein kinase B (Akt) pathway [158]. Retrorsine is shown to induce rats’ HSOS in 14-week exposure, with TGF-β1/Akt phosphorylated activation [157]. Whereas single exposure results in transient, self-limiting fibrosis, repeated PAs exposure elicits persistent and robust fibrosis. Notably, the chronological order between fibrosis and inflammation in PA-induced liver injury remains unclear.

### 4.5. Ferroptosis

Distinct from apoptosis, ferroptosis is an iron-dependent novel form of regulated cell death, driven by excessive lipid peroxidation. It can be regulated by Cystine/GSH/Gpx4 axis [159]. Acyl-CoA synthetase long-chain family member 4 (ACSL4) is essential for ferroptosis execution and determines cellular susceptibility to ferroptosis, of which upregulation is consistent with enhanced ferroptosis signal. Transferrin receptor protein 1 (TFR1) primarily mediates iron ion uptake by holo-transferrin. Mammalian target of rapamycin (mTOR, a serine/threonine kinase) regulates protein synthesis and can inhibit cystine deprivation-induced ferroptosis [159]. Previous findings may link PA-induced lipid dysregulation and GSH depletion to potential ferroptosis processes, though conclusive evidence remains lacking. Liu et al. provide that oral administration of *Emilia sonchifolia* (L.) extract at 13.72 g/kg for 14 days exhibits activation of ferroptosis pathways across metabolomic, transcriptomic, and proteomic analyses in the liver tissues of mice [160]. Specifically, it upregulates ACSL4 mRNA and protein levels, increases TFR1 and mTOR abundance, and elevates MDA content [160]. Oral administration of retrorsine at 100 mg/kg for 48 h significantly induces hepatic 4-hydroxynonenal staining intensity (lipid peroxidation biomarker) in rats [161]. Transmission electron microscopy reveals morphological hallmarks of ferroptosis, such as mitochondrial shrinkage, increased membrane density, and loss of cristae structure [161]. Moreover, mRNA levels of ACSL4 and prostaglandin-endoperoxide synthase 2 are upregulated, whereas Gpx4 is downregulated. In the same investigation, ferroptosis inhibitor ferrostatin-1 cotreatment partially alleviates liver injury, and it confirms the participation of ferroptosis [161].

### 4.6. Autophagy

Autophagy, an evolutionarily conserved cellular stress response mechanism for maintaining homeostasis, is dynamically regulated during liver injury mediated by certain PAs. Autophagic activity is not consistently activated or suppressed in PA-related HILI. Senecionine, clivorine, or seneciphylline exposure (3.125–12.5 μM for 24 h) can dose-dependently elevate the expression of microtubule-associated protein light chain 3 (LC3)-II/LC3-I ratio and the formation of mature autophagosomes in Huh-7.5 cells [162]. In primary mouse hepatocytes, senecionine (20 μM for 6 h) selectively activates autophagy for mitochondria after mitochondrial damage, while maintaining overall autophagy flux, representing a self-protective mechanism that mitigates PA-induced apoptosis [132]. Similarly, retrorsine treatment at 25–100 μM for 24–48 h has been shown to trigger oxidative stress and autophagy activation in primary rat hepatocytes [99]. In contrast, oral administration of *Gynura segetum* extract at 30 g/kg/d for 5 weeks significantly downregulates the protein expression of LC3, autophagy-related 12 (Atg12), and cytoplasmic polyadenylation element binding protein 4, and exacerbates apoptosis in the livers of mice [39]. Inhibition of autophagy markedly aggravates senecionine-induced (at 6.25 μM for 24 h) apoptosis, whereas the activation of autophagy attenuates cytotoxicity in Huh-7.5 cells [162]. In conclusion, to determine the real role and threshold of cellular fates under the complex crosstalk, a specific evaluation of liver injury induced by certain PAs is required.

Research shows that retrorsine treatment at 50 μM for 24 h significantly enhances the expression of phosphorylated ERK, JNK, and p38 proteins, and then promotes the expression of Beclin 1 and LC3-II proteins in cascade. In primary rat hepatocytes, it triggers the formation of autophagosomes, and these changes can be reversed by corresponding MAPK inhibitors [99]. Additionally, monocrotaline treatment at 300 and 400 μM for 36 h activates the canonical PI3K/Akt/mTOR pathway to drive autophagy in primary rat hepatocytes [163]. All of these findings indicate that PA-mediated autophagy is largely attributed to the activation of the MAPK pathway and PI3K/Akt/mTOR pathway.

### 4.7. Cell Cycle Arrest and DNA Damage

PAs can interfere directly with cell cycle checkpoints or induce persistent DNA damage that overwhelms repair pathways, and they contribute to mutagenesis and carcinogenesis [164]. Several hepatotoxic members of PAs (such as retrorsine, monocrotaline, and lasiocarpine) have been shown to induce G2-phase arrest in hepatocytes [165,166]. Differing from previous observations, it is reported that riddelliine treatment at 2.5 mg/kg for a continuous 8 days can induce S-phase arrest in both parenchymal and non-parenchymal mouse hepatocytes [167]. According to the “Golgi blockade” hypothesis, the mitosis regulatory sensor-Golgi is responsible for the cell cycle arrest prior to M-phase [168,169]. Golgi fragmentation and mitosis are often promoted by phosphorylation of the Ser site in the cis-Golgi scaffolding protein and Golgi matrix protein 130. Scaffold/raft protein caveolin-1 regulates mitosis by binding to its respective receptors and signaling molecules [169]. Monocrotaline treatment at 100 μM for 48 h significantly induces cell cycle arrest prior to Golgi fragmentation but subsequent to Golgi matrix protein 130 phosphorylation. Meanwhile, monocrotaline also results in caveolin-1 failure assembly and is trapped within lighter-density Golgi fractions, which then disrupts the subcellular location of diverse signaling molecules (e.g., signal transducer and activator of transcription 3 and ERK1/2) [169,170].

The tumor suppressor protein p53, a critical regulator of genomic stability, governs cell cycle progression and programmed cell death. The mutations of p53 serve as early molecular events in carcinogenesis and it is detected in riddelliine-exposed (at 1.0 or 2.5 mg/kg per day; 5 doses/week for 6 weeks) rat liver tissues [167]. Transcriptomic and proteomic profiling of rat livers subjected to retrorsine at 3.3 mg/kg for 28 days can induce the enrichment of chemical carcinogenesis–DNA adduct pathways and marked DNA damage [171].

As a malignant outcome of cycle arrest and DNA damage, the carcinogenic potential of PAs has been substantiated in numerous animal models of dietary PA exposure [95,172,173,174,175,176]. One plausible explanation is that compensatory DNA synthesis or regenerative hyperplasia triggered by DNA adducts may facilitate the fixation of p53 mutations and other genomic alterations [177].

### 4.8. Metabolic Disorder

PAs hepatotoxicity requires CYP-mediated bioactivation, yet PAs also conversely regulate metabolic enzymes. For instance, gestational exposure induces metabolism-related sensitivity in offspring. Monocrotaline exposure to the maternal at 20 mg/kg/d from gestation day 9 to 20 upregulates CYP3A protein level in female offspring, while it downregulates CYP3A in males [178]. This implicates the dysregulation of the upstream transcriptional regulator, pregnane X receptor (PXR) [178]. Dai et al. further demonstrate that retrorsine treatment at 20 mg/kg for 11 days can promote the protein expression of PXR in the nucleus, and significantly upregulate the expression of CYP3A1, CYP3A2, CYP3A9, and CYP3A18 mRNA levels in the livers of pregnant rats. This causes a higher susceptibility for retrorsine-induced liver injury in female offspring due to the higher amount of PA–protein adducts in the liver [179]. Such differences in metabolic disorders are likely driven by the patterns of release of sex hormones and growth hormones. Male offspring exhibit androgen receptor-mediated blockade of PXR activation and enhanced tolerance to the hepatotoxic effects of PAs [179].

Disorders of bile metabolism have garnered attention as a clinical manifestation in HSOS. Oral administration of senecionine at 60 mg/kg for 24 h can significantly inhibit the expression of genes related to bile acid absorption (such as solute carrier family (Slc)10a1, Slc22a7, and Slc22a21), bile acid excretion (such as ATP-binding cassette subfamily B (ABCB) 1, Abcb11, Abcb4, and Abcc2), and bile acid synthesis (such as CYP7a1) in mice [64]. Of note, exposure to four PAs (echimidine, senecionine, heliotrine, or senkirkine) at 35 and 70 μM for 24 h or 14 days can both disrupt the mRNA expression related to multiple transporters and bile acid enzymes, and impair hepatocellular excretory function in HepaRG cells [180]. Senecionine treatment at 35 mg/kg for 36 h disrupts several pathways in bile metabolism, such as primary bile acid biosynthesis and bile secretion in rats [181]. Farnesoid X receptor (FXR) is a more upstream core target, with its interaction with PAs based on a prototypical conformation. This view is supported by indirect evidence from computational simulations and experiments in mouse primary hepatocytes [182]. Oral administration of senecionine at 50 mg/kg significantly reduces FXR activity in mice and promotes hepatic bile acid biosynthesis via the FXR–small heterodimer partner signaling pathway [182].

### 4.9. Others

Multi-omics analyses have markedly advanced the understanding of PA-induced hepatotoxic mechanisms. Non-coding microRNAs can transcriptionally silence specific genes, and the miRNAome expression profile elucidates complementary insights into transcriptional regulation. Huang et al. conduct an integrative mRNA-microRNA analysis in mice to predict microRNA targets [183]. Following monocrotaline treatment at 270 and 330 mg/kg for 48 h, eight microRNA targets are identified and annotated to the “phagosome” pathway, which mediates post-inflammatory immune processes in the liver [183]. Enge et al. identify another nine time-dependent differentially expressed microRNAs in senecionine-treated (at 35 μM for 24 h) HepaRG cells. IPA identifies the primary hepatotoxicity pathways as “hepatic proliferation” and “hepatocarcinogenesis” [184].

The existence of the gut–liver axis in PA-induced HSOS has been supported by multi-omics evidence. Microbiome and metabolome profiles of mice exposed to monocrotaline (at a single dose of 750 mg/kg; oral administration) reveal reductions in Firmicutes, Lachnospiraceae, Phascolarctobacterium, Coprococcus, Roseburia, and Clostridium_XlVb, which are associated with decreased levels of short-chain fatty acids such as butyrate [185]. Decreased butyrate promotes M1 macrophage polarization and exacerbates HSOS in mice [185]. Tryptophan metabolism represents another critical substance of the gut–liver axis. Monocrotaline treatment at 45–90 mg/kg for 48 h is shown to disrupt microbiota and suppress the production of indole derivatives. This ultimately impairs aryl hydrocarbon receptor (AhR) activation and intensifies oxidative stress via the AhR/Nrf2 axis [111].

## 5. Detoxification Strategy

### 5.1. Targeted Treatment of Oxidative Stress and Mitochondrial Damage

Therapeutic strategies targeting oxidative stress and mitochondrial dysfunction have emerged as key approaches to alleviate hepatotoxicity. Early experiments have demonstrated the existence of GSH-DHP adducts in rat bile and plasma, and NAC-DHP in excreta [186,187]. Meanwhile, GSH supplementation at 2.0 mM inhibits CYP-mediated PAs bioactivation in hepatic microsomes [25]. Accordingly, this implies that GSH-related substances accelerate PAs detoxification and excretion. For instance, pretreatment with S-adenosyl methionine (a cysteine precursor) at 5 μM for 2 h ameliorates clivorine-induced (at 5–100 μM for 48 h) hepatotoxicity [115]. Similarly, antioxidant NAC treatment (at 5 mM on hour 24) markedly attenuates clivorine-induced (at 50 μM for 48 h) DNA fragmentation, depletion of intracellular GSH, and activation of mitochondrial apoptotic signaling in human L-02 cells [116]. NAC treatment at 2.5 mM for 24 h also protects primary hepatocytes by suppressing autophagy [99]. Oral supplementation with selenium (a constituent element of GPx) at 0.25 mg/kg or vitamin E (a lipid-soluble antioxidant) at 200 mg/kg for 7 days partially reverses monocrotaline-induced liver damage by restoring biochemical GSH levels and improving liver structure in rats [188]. Mitochondrial protein thiol oxidation is another PA-induced mechanism contributing to apoptosis. Dithiothreitol (at 10 mM for 90 min) maintains the reduced state of thiols in mitochondrial membrane proteins and prevents Cyt C release in monocrotaline-treated primary rat hepatocytes [189]. Phosphocreatine mitigates the hepatotoxicity of the PA-containing plant *Gynura segetum* via the SIRT3-SOD2–mitochondrial pathway in L-02 cells and C57BL/6J mice [105]. This highlights the importance of counteracting mitochondrial oxidative stress as a potential therapeutic target, and it is further validated by the protective effects of Mito-TEMPO (a specific mitochondrial superoxide scavenger) at 50 μM for 8 h [105]. Moreover, intraperitoneal injection of phosphocreatine at 50 mg/kg for 3 days also reduces monocrotaline-induced ER stress by inhibiting proline/serine-rich coiled-coil 1 (PSRC1, a microtubule-associated protein regulating spindle dynamics, cell proliferation, and immune responses), and it reveals a novel detoxification mechanism distinct from the energy buffer of ATP homeostasis [190]. Oral administration of cerium oxide nanoparticles at 0.01 ng/kg restores the decreases in GSH content and GR, GPX, GST, CAT, and SOD activities caused by monocrotaline exposure [191].

### 5.2. Natural Product Therapeutic Approaches

Natural product-derived antioxidants exhibit potent hepatoprotection against PA-induced injury. Quercetin treatment (40–90 mg/kg, oral administration) protects against clivorine-induced hepatic apoptosis in mice by inhibiting caspase-3 activation, reducing 4-hydroxynonenal immunoreactivity, and reversing the upregulation of oxidative stress–associated genes, including heme oxygenase-1/2 and flavin-containing monooxygenase 5 (FMO5) [192]. Similarly, oral administration of 18β-glycyrrhetinic acid at 50 mg/kg/d for 5 days alleviates retrorsine-induced oxidative stress in rats by activating Nrf2-dependent ARE via PI3K/Akt/glycogen synthase kinase-3 signaling cascade [110,193]. Quercetin or baicalin (two doses of oral administration, 40 mg/kg on 6 and 30 h) activates TLR/NF-κB signaling, enhances Nrf2 activity, and modulates the MAPKs and PI3K/Akt pathways, which ameliorate HSOS symptoms in rats [109]. Furthermore, two oral doses of hyperoside (20, 40, or 80 mg/kg at 6 and 30 h) enhance autophagy and preserve lysosomal function through activation of transcription factor EB (TFEB) [194]. Hyperoside also attenuates *Gynura japonica* alkaloid-induced hepatotoxicity [194]. Furthermore, hyperoside inhibits mTOR complex 1, which drives TFEB nuclear translocation and peroxisome proliferator-activated receptor–gamma coactivator 1α, and it stabilizes mitochondrial function via the autophagy–lysosome pathway [194]. This mechanism may underlie the absence of reported hepatotoxicity for certain PA-containing plants, likely due to the counteracting effect between PAs and hyperoside. In addition, bile duct-targeted traditional remedies also confer hepatoprotection. Bear bile powder treatment at 250, 500, or 1250 mg/kg (two doses gavaged on days 1 and 4) significantly inhibits the expression of TGF-β and pro-inflammatory factor levels, reduces bile acid stasis, and finally reverses senecionine-mediated HSOS [195]. FXR regulates bile acid synthesis, uptake, and excretion via SIRT1-mediated acetylation and deacetylation. Oral administration of chlorogenic acid at 20–80 mg/kg (two doses at 6 and 30 h) promotes SIRT1-mediated FXR deacetylation to restore bile acid homeostasis [196]. Additionally, *Alismatis rhizoma* water extract, Danning tablet powder, Liquorice extract, Schisandrol A, and Alisol B 23-acetate have been reported to exert protective effects against PA-related HILI.

### 5.3. Small-Molecule Inhibitors/Agonists and Others

Among small-molecule therapeutics, oral administration of FXR agonist obeticholic acid at 20 mg/kg (one pretreatment dose for 3 days or two post-treatment doses on 6 and 30 h) reverses senecionine-induced (single oral dose at 50 mg/kg) hepatic injury [182]. Few studies address PA-induced hepatic fibrosis, but prednisone treatment (oral administration, at 5 mg/kg/d for 30 days) suppresses the mRNA expression of connective tissue growth factor, TGF-β1, TNF-α, and NF-κB, which significantly ameliorates fibrosis in PA-induced (administered with *Gynura segetum* extract at 30 g/kg/d for 30 days) liver injury [197]. As CYP3A4 drives the metabolic activation of most PAs, its inhibition represents a major therapeutic target. Oral administration of ritonavir (a potent CYP3A4 inhibitor) at 30 mg/kg significantly decreased the levels of serum ALT, TBA, and pyrrole–protein adducts and alleviated hepatic sinusoidal congestion and necrosis in liver tissues exposed to *Gynura japonica* extracts (at 8 mg/kg orally) in rats [198]. Hepatic transport systems also contribute to PA-specific accumulation. Pretreatment with organic cation transporter 1 (OCT1) inhibitors (i.e., D-tetrahydropalmatine or quinidine) has been shown to effectively protect primary rat hepatocytes from PA-induced damage [199,200]. Wang et al. identify TSP1 as a key differentially expressed protein in the early phase of senecionine-induced (at 50 mg/kg orally) HILI. The two oral doses of specific TSP1 inhibitor leucine–serine–lysine–leucine peptide (at 50 mg/kg on 0.25 and 6 h) attenuate sinusoidal endothelium damage in mice [76]. Other therapeutic molecules (e.g., anticoagulant enoxaparin) and gut microbiota-derived metabolites (e.g., indole-3-acetaldehyde, indole acetic acid, and sodium butyrate) also manifest potential benefit in ameliorating PA-related HILI.

Table 2 provides an overview of studies from the past two decades on attenuation strategies. Owing to the multifactorial mechanisms in PA-related HILI, a monotherapeutic agent targeting a single mechanistic pathway is insufficient to resolve complex hepatotoxicity. Therefore, combination therapy or broad-spectrum agents should be developed as a future paradigm for effective intervention.

## 6. Regulation and Prevention in China

Given the widespread presence and hazard potential of PAs in natural products, herbal medicines, and dietary supplements, several governments and organizations have implemented corresponding regulatory frameworks. The European Commission updated the maximum permitted levels of PAs in foodstuffs in 2023, specifying that the PA content in herbal-containing food supplements must not surpass 400 μg/kg [206]. The Chinese Pharmacopoeia prescribes that the amount of adonifoine (a representative PAs) in *Senecio scandens* shall not exceed 0.004% [207]. China’s agricultural or food sector has not yet established the daily maximum consumption of PAs or the detection threshold for such compounds in relevant products.

The Centre of Food Safety of Hong Kong has performed dietary exposure assessments of PAs in the local population. The average consumption ranges from 0.0003 to 0.0043 μg/kg/d, which is markedly lower than 0.0182 μg/kg/d. It indicates that the health issue of PAs for the public is deemed negligible [208]. The Centre of Food Safety recommends that food manufacturers refer to guidelines for managing PA-containing weeds in food and feed to enhance the planting, harvesting, and cleaning methods of associated products [208,209]. However, it is also pointed out that the full eradication of PA-containing plants is neither practical nor ecologically desirable.

Notably, the European Union has implemented a Rapid Alert System for Food and Feed, which enables efficient information exchange among member states and empowers the food sector to respond promptly to public health risks [210]. Chinese authorities should strengthen the supervision and control of PA-containing products. This entails defining upper limits for residents’ consumption, setting a statutory detection threshold for PAs, and developing a rapid early-warning system for PAs contamination, to guarantee swift reaction and management of such crises and protect public health.

## 7. Conclusions and Prospects

**Figure 6:**
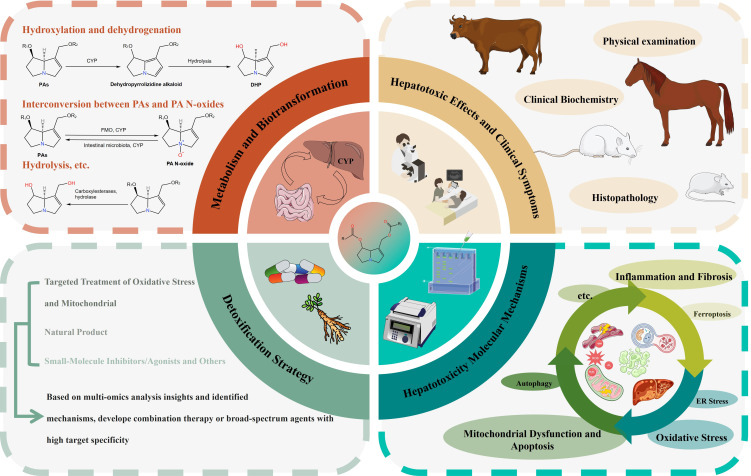
An overview of the biotransformation, hepatotoxic effects, molecular mechanism, and detoxification strategy of PAs.

As widely distributed plant-derived toxins, PAs pose significant risks upon ingestion via herbal medicines, food products, or environmental exposure. Despite comprehensive research on PA-related HILI, adverse event reports and incidents of food chain contamination continue to emerge. This review concisely outlines the toxicological basis of PAs and emphasizes recent advances in PA-induced cell death patterns and upstream regulatory signaling (Figure 6). PA-related HILI encompasses a cascade of events, including oxidative stress, mitochondrial dysfunction, apoptosis, ER stress, inflammation, fibrosis, autophagy, ferroptosis, necrosis-like injury, with the participation of key signaling pathways such as Nrf2, MAPK, PERK, TLR4, NF-κB, TGF-β, p53, FXR, and PXR. Apoptosis constitutes the predominant cell death pattern, while multi-omics analyses have validated the involvement of ferroptosis and the liver–gut axis in PA-related HILI. Despite advances in understanding these toxic pathways, effective clinical interventions and detoxification strategies remain limited.

Currently, several priority areas warrant further investigation. First, the crosstalk among different cell death pathways (e.g., apoptosis, ferroptosis, and autophagy) in PA-induced liver injury should be systematically dissected, ideally using in vivo models with genetic or pharmacological modulators. Second, the role of gut microbiota in metabolizing PAs and modulating hepatotoxicity represents an underexplored avenue that could unveil novel therapeutic targets. Third, there is an urgent need to develop sensitive and specific biomarkers for early diagnosis of PA exposure and liver damage, including pyrrole-protein adducts, circulating miRNAs, and metabolomic signatures. Fourth, future research should focus on designing safe and effective detoxification strategies, such as natural antioxidants (e.g., NAC and vitamin E) that counteract oxidative stress, inhibitors of CYP-mediated bioactivation, or inducers of phase II detoxifying enzymes (e.g., Nrf2 activators). Fifth, advanced technologies including organ-on-a-chip, single-cell sequencing, and CRISPR-Cas9 screening could help unravel inter-individual susceptibility and sex differences in PA-induced hepatotoxicity. Finally, stronger regulatory frameworks and risk assessment models are essential to monitor PA contamination in food, herbal products, and the environment, thereby reducing global public health risks.

Based on the characterization of PAs’ hepatotoxic targets, detoxification strategies can be categorized into three categories, including therapeutics targeting oxidative stress and mitochondrial damage, natural product therapies, and small-molecule targeted drugs. Nevertheless, combinatorial or broad-spectrum interventions and rigorous clinical evaluation are still necessary to mitigate the PA-related HILI. The horizontal transfer of PAs across water, soil, and plants elevates environmental exposure risks. Chronic low-level exposure in particular deserves scrutiny for bioaccumulation and persistent metabolite formation, a profile clearly distinct from acute toxicity. Long-term monitoring using high-content screening is essential to comprehensively characterize accumulated adverse alterations at the molecular level. Beyond CYP-mediated bioactivation, the contribution of parent PAs to hepatotoxicity merits careful consideration, even in the absence of direct lethality. These insights will support improved monitoring of PAs in food, plant-based products, and the environment, while concurrently advancing the elucidation of hepatotoxic mechanisms and the evolution of targeted therapeutic strategies.

## Figures and Tables

**Table 1:** A summary of PA-related HILI.

Plant	Animal	Exposure Method	PAs Content and Consumption	Biochemical, Pathological Characteristics, or Clinical Symptoms	Reference
*Senecio pampeanus*	Cattle/Hereford heifer	Grazing in native grassland, for a period of 5 months.	Each gram of plant contains 0.4 mg total PAs (including Florosenine, doronine, otosenine, desacetyldoronine).	Biochemical characteristics: ↑ AST; ↓ TP, ALB. Pathological characteristics: Fibrosis, bile duct proliferation, and hepatomegaly.	[28]
*Crotalaria spectabilis*	Cattle/Nellore cattle	Grazing in native grassland for 20 days.	Monocrotaline.	Clinical symptoms: Rough hair coat, anorexia, isolation from the herd, weight loss, jaundice, recumbency, and death. Pathological characteristics: Centrilobular and bridging hepatocyte loss, sinusoids dilation, fibrosis, hepatomegalocytosis, and cholestasis.	[29]
*Gynura japonica* (Thunb.) Juel	Human	Oral administration of *Gynura japonica* decoction or herb wine for 7–90 days.	/	Clinical symptoms: Ascites, abdominal distension, jaundice and hepatomegaly, with Rucam score between 6 and 10. Biochemical characteristics: ↑ AST, ALT, ALP, TBIL. Pathological characteristics: Hepatic sinusoidal dilation, hepatocyte necrosis, hemorrhage, and congestion.	[30]
	Sprague-Dawley rat	Oral administration of decoction at 1500 mg/kg/d for 7–14 days.	Senecionine at 28.2 mg/kg, seneciphylline at 110.38 mg/kg, seneciphylline *N*-oxide at 43.9 mg/kg, and jacobine *N*-oxide at 20 mg/kg in decoction.	Biochemical characteristics: ↑ AST, ALT, ALP, DB, TBIL; ↓ ALB. Pathological characteristics: Granulomas, inflammatory cell infiltration, congestion, sinusoidal dilation, and cholestasis.	[31]
	Mouse	Single oral dose of extracts at 0.5, 1, and 2 g/kg, or multiple oral doses at 0.5 g/kg/d for 1–8 weeks.	Extract contains 4.65% total PAs (including senecionine and its *N*-oxide at 2.72%, seneciphylline and its *N*-oxide at 0.33%).	Pathological characteristics: Inflammatory cell infiltration, apoptosis, sinusoidal hemorrhage, hepatocyte necrosis, hepatocellular balloon-like lesions, endothelial injury of central venules, and collagen enrichment.	[32]
	Mouse	Single oral administration of total extracts at 1.0 g/kg or total alkaloids at 60 mg/kg.	Each gram of total alkaloids contains 1.74–7.42 mg total PAs (including seneciphylline *N*-oxide, senecionine *N*-oxide, and spartioidine, etc.).	Biochemical characteristics: ↑ ALT, AST, TBIL, TBA. Pathological characteristics: Hepatic sinusoidal hemostasis, deposition of erythrocytes, endothelial injury of central venules, destruction of hepatocytes, inflammatory cell infiltration in hepatic lobules, and collagen enrichment at sinusoidal and venous lumens	[33]
*Jacobaea vulgaris* Gaertn	Cow/Holstein cow	Oral administration for 7, 14, 21, and 28 days.	The exposure to total PAs (including Jaconine, Jaconine *N*-oxide, Seneciphylline *N*-oxide, etc.) at the level of 4184.4 mg/kg diet.	Biochemical characteristics: ↑ AST, GGT; disturbance of ALT, TP, ALB, TBIL, ALP. Pathological characteristics: Hepatocyte nucleus enlargement and macrophage infiltration.	[34,35]
*Gynura segetum*	Human	Oral administration of extract at 100 mL on day 3 before the onset of disease.	/	Biochemical characteristics: ↑ DB, AST, ALT; ↓ TP, ALB. Clinical symptoms: Abdominal distension, weakness, poor appetite, intermittent vomiting, and sinusoidal dilation. Pathological characteristics: Hepatocellular hydropic degeneration, necrosis, and inflammatory cell infiltration. The case meets Nanjing criteria.	[36]
	Human	Oral administration of *Gynura segetum* for 8–90 days.	/	Biochemical characteristics: ↑ AST, ALT; GGT, TBIL. Clinical symptoms: Enlarged liver, ascites, abdominal pain, abdominal distension, nausea, vomiting and jaundice. Pathological characteristics: Hepatic lobules, central vein fibrosis, hepatic sinusoids dilatation and congestion, hepatocytes atrophy, and lymphocytes and foam cells infiltration. All cases meet Nanjing criteria.	[37]
	Rat	Oral administration of decoction at 7.5 or 15 g/kg/d for 21 days.	It contains seneciphylline, senecionine, and seneciphylline *N*-oxide.	Biochemical characteristics: ↑ AST, ALT. Pathological characteristics: Disordered arrangement of hepatocytes, necrosis, vacuolar degeneration, sinusoidal hemorrhage, and decreased diversity of gut microbiota.	[38]
	Mouse	Oral administration of decoction at 30 g/kg/d for 5 weeks.	It contains seneciphylline, senecionine, and seneciphylline *N*-oxide.	Biochemical characteristics: ↑ AST, ALT; ↓ Triglyceride. Clinical symptoms: Abdominal distension, ascites, and weight gain. Pathological characteristics: Lobule structure destruction, hepatocyte nucleus pyknosis, inflammatory cell accumulation in the portal, fibrosis, fatty degeneration, and sinusoidal congestion.	[39]
*Eupatorium fortune* Turcz	Mouse	Oral administration of total alkaloids at 25 mg/kg/d for 4 weeks.	Total alkaloids contain 29.55% echinatine *N*-oxide, 17.28% echinatine, and 14.47% lycopsamine *N*-oxide.	Biochemical characteristics: ↑ AST, ALT, GST, LDH, GSSG/GSH. Pathological characteristics: Hepatocyte nucleus and cytoplasm enlargement, vacuolation, and hepatocyte apoptosis.	[40]
*Senecio oxyphyllus*	Cow/Holstein cow	Oral administration of dry plant at 4 g/kg/d for 24 days.	Each gram of dry plant body contains 2.6 mg retrorsine.	Clinical symptoms: Lethargy, weight loss, abdominal distension, and ascites. Pathological characteristics: Hepatomegalocytosis, fibrosis, hepatocyte necrosis and degeneration, and bile duct epithelial proliferation.	[41]
*Gynura rhizoma*	Mouse	Oral administration of extracts at 1.0 g/kg/d for 40 days.	Each gram of extracts contains 15.9 mg Senecionine, 15.9 mg seneciphylline, and 25.9 mg seneciphylline *N*-oxide.	Clinical symptoms: Piloerection, decreased movement, abdominal distension, and ascites. Pathological characteristics: Lobular structure damage, coagulative necrosis, sinusoid hemorrhage, endothelial damage, and fibrosis.	[42]
*Senecio brasiliensis*	Human/woman	Oral administration of a homemade tea of *Senecio brasiliensis* for 20 days.	/	Clinical symptoms: Jaundice, ascites and bilateral pleural effusion, with a RUCAM score of 6. Biochemical characteristics: ↑ AST, ALT, TBIL, ALP, GGT. Pathological characteristics: Lobular center vein sclerosis.	[43]
	Human	Oral administration of *Senecio brasiliensis* steeping in water, decocting in water or soaking in wine.	/	Clinical symptoms: Abdominal distension, poor appetite, jaundice, hepatomegaly, ascites, and edema of both lower limbs, with a RUCAM score between 4 and 8. Biochemical characteristics: ↑ AST, ALT, TBIL. Pathological characteristics: Lobular center vein sclerosis.	[44]
	Horse	Free feeding in the enclosure.	/	Clinical symptoms: Anorexia, weight loss, dysmetria, proprioceptive deficit, and signs suggestive of colic. Pathological characteristics: Megalocytosis, fibrosis, and bile duct hyperplasia.	[45]
	Horse	Grazing in native grassland for 8 months.	/	Biochemical characteristics: ↑ GGT, ALP; ↓ ALB. Pathological characteristics: Fibrosis, coagulative necrosis, biliary duct hyperplasia, megalocytosis, and cholestasis.	[46]
	Cattle	Grazing in native grassland.	/	Clinical symptoms: Weight loss, weakness, diarrhea, edema, photophobia, tearing, salivation, and neurological symptoms Pathological characteristics: Vacuolation, hepatomegalocytosis, bile duct proliferation, and fibrosis.	[47]
*Crotalaria incana*	Cattle	Grazing in native grassland for 6 months.	Each gram of plant contains 10–870 µg total PAs (including Usaramine and usaramine *N*-oxide).	Clinical symptoms: Depression, weight loss. Pathological characteristics: Bile duct proliferation, fibrosis, vacuolation, and hepatomegalocytosis.	[48]
*Anchusa boraginaceae*	Human/man	Oral administration of the extract via drinking water for 14 days.	/	Clinical symptoms: Nausea, fatigue, jaundice. Biochemical characteristics: ↑ AST, ALT, ALP, GGT, and TBIL. Pathological characteristics: Portal inflammation, feathery degeneration, cholestasis.	[49]
*Senecio grisebachii*	Cow/Holstein or Holstein × Jersey cow	Oral administration of 30% decoction at 15 g/kg per 48 h for 6 days, 24 g/kg per 48 h for 10 days, or 45 g/kg per 48 h for 20 days.	It contains 0.29% total PAs (senecionine, seneciophylline, retrosine, etc.).	Clinical symptoms: Depression, anorexia, weight loss, abdominal pain, tenesmus, submandibular edema, colic, and lateral recumbency. Biochemical characteristics: ↑ AST, ALT, and GGT. Pathological characteristics: Bile duct proliferation, hepatocyte necrosis, hepatomegalocytosis, fibrosis, granular degeneration, and vacuolation.	[50]
*Senecio candicans*	Rat	Oral administration of extracts at 250–750 mg/kg/d for 90 days.	Each gram of the leaf extract of Senecio candicans contains 280 μg total PAs.	Biochemical characteristics: ↑ AST, ALT, ALP, and LDH. Pathological characteristics: Monocyte infiltration, nucleus enlargement, hepatocyte architecture loss, vessel dilation, and hepatocyte hydropic change.	[51]

Note: / indicates no data; ↑ indicates upregulation; ↓ indicates downregulation.

**Table 2:** A Summary of PA-related HILI attenuating strategies.

PA-Induced Liver Injury Models	Treatment	Therapeutic Effect	Reference
Treatment of clivorine at 5–100 μM for 48 h in L-02 cells.	Pretreatment of s-adenosyl methionine at 5 μM for 2 h.	It significantly rescues cytotoxicity in a concentration-dependent manner and enhances GSH content.	[115]
Intraperitoneal injection of monocrotaline at 300 mg/kg on day 0 in Wistar albino rats.	Oral administration of selenium at 0.25 mg/kg or vitamin E at 200 mg/kg for 7 days.	It improves the serum GSH level, liver histopathological score, and partially rescues apoptosis, GPx, and CAT activities.	[188]
Treatment of monocrotaline at 5 mM for 90 min in rat primary hepatocytes.	Pretreatment of dithiothreitol at 10 mM for 15 min.	It prevents cytotoxicity and loss of intracellular ATP, inhibits the oxidation of protein thiol groups, and evaluates GSH.	[189]
Intraperitoneal injection of monocrotaline at 10 mg/kg on day 4 in rats.	Intraperitoneal injection of cerium oxide nanoparticles at 0.01 ng/kg on days 1, 3, 5, and 7.	It restores GSH content, and GR, GPX, GST, CAT, and SOD activities.	[191]
Oral administration of *Gynura segetum* extraction at 30 g/kg/d for 30 days in mice.	Oral administration of prednisone at 5 mg/kg/d for 30 days.	It partially improves liver endothelial damage, necrosis, fibrosis, and inflammation, and reduces the expression of TGF-β1, CTGF, TNF-α, and NF-κB p65.	[197]
Treatment of monocrotaline at 50–200 μM or retrorsine at 10–50 μM for 48 h in rat primary hepatocytes.	Treatment of D-tetrahydropalmatine (OCT1 inhibitor) at 40 μM or quinidine (OCT1 inhibitor) at 20 μM for 48 h.	It attenuates PA-induced hepatotoxicity.	[199,200]
Oral administration of clivorine at 210 mg/kg after quercetin pretreatment in mice.	Oral administration of quercetin at 40, 60, and 90 mg/kg/d for 7 days.	It decreases serum ALT and AST activity and TB level, reverses intrahepatic hemorrhage, destruction of liver structure, GSH depletion, lipid peroxidation, and apoptosis, which involves regulation of mRNA level, such as Fmo5, Sod2, Hmox2, Hmox1, Cyp2b10, Cyp1b1, Hspa5, and Hspa1l.	[192]
Oral administration of monocrotaline at 90 mg/kg on hour 0.	Oral administration of quercetin or baicalein at 40 mg/kg on hours 6 and 30.	It inhibits HSOS symptoms, eliminates monocrotaline-induced NFκB p65 nuclear translocation, Egr1 activation, upregulation of MAPKs and PI3K/AKT signaling cascade, and promotes Nrf2 activation.	[109]
Oral administration of *Gynura segetum* extracts at 30 g/kg/d for 3 weeks in mice.	Oral administration of phosphocreatine at 50 or 100 mg/kg/d for 3 weeks.	It improves liver function and pathological damage, as well as oxidative stress states, which involves the SIRT3-SOD2–mitochondrial ROS pathway and apoptosis.	[105]
Oral administration of *Gynura rhizoma* at 100 μL 30 g/kg/d for 40 days in mice.	Oral administration of Danning tablet powder at 30 g/kg for 40 days.	It improves symptoms of HSOS, including sinusoidal/subendothelial hemorrhage and fibrosis, and it involves inhibition of TGF-β/p-Smad3 pathway and inflammation-related factors.	[201]
Oral administration of senecionine at 50 mg/kg/d on day 6 in mice.	Oral administration of *Alismatis rhizoma* water or ethanol extract at 18 g/kg/d on days 1–5.	It improves pathological liver damage and serum bile acid levels, involving restoring bile excretion and metabolic detoxification of bile acids.	[202]
Oral administration of *Gynura japonica* extracts at 8 mg/kg in rats.	Single oral dose of ritonavir (CYP3A4 inhibitor) at 30 mg/kg 1 h before modeling.	It reduces pathological sinusoidal hemorrhage, spotty necrosis, vacuolization, and bile acid disorders.	[198]
Oral administration of retrorsine at 40 mg/kg 2 h after the last dose of the drug in rats.	Oral administration of liquorice extract at 500 mg/kg/d or 18β-glycyrrhetinic acid at 50 mg/kg/d for 5 days.	It dose-dependently improves cellular necrosis, hemorrhage, and serum liver injury markers, which involves oxidative stress responses associated with Nrf2-ARE and upstream PI3K/Akt/GSK3β signaling pathway.	[110,193]
Oral administration of senecionine at 50 g/kg in mice.	Intraperitoneal injection of leucine–serine–lysine–leucine peptide (TSP1 inhibitor) at 50 mg/kg on 0.25 and 6 h.	It reduces serum liver injury markers and damage of sinusoidal endothelium, involving blocking the action of TSP1.	[76]
Oral administration of senecionine at 50 g/kg i.g. on day 3 in mice.	Oral administration of bear bile powder at 250/500/1250 mg/kg on days 1 and 4.	It improves histological, serum symptoms of HSOS, bile acid homeostasis, inflammation, and fibrosis.	[195]
Oral administration of monocrotaline at 90 g/kg i.g. on day 1 in rats.	Subcutaneous injection of enoxaparin at 8 mg/ kg/d on days 2–8.	It alleviates sinusoidal dilatation and endothelial hemorrhage, and improves serum indicators, which may involve the suppression of oncostatin M expression (a member of the pleiotropic interleukin family).	[203]
Oral administration of senecionine at 150 μmol/kg on day 7 in mice.	Oral administration of schisandrol A at 116 μmol/kg on days 1–7.	It improves hepatic necrosis, sinusoidal congestion, and serum indicators, involving the inhibitory effect on enzymes related to the metabolic activation of PAs.	[204]
Oral administration of monocrotaline at 90 mg/kg on day 6 in SD rats.	Oral administration of indole-3-acetaldehyde or indole acetic acid at 20 mg/kg on days 1–5.	It eliminates PA-induced sinusoidal congestion, necrosis, and endothelial injury, which involves AhR/Nrf2 signaling-mediated alleviation of oxidative stress.	[111]
Single oral dose of total alkaloid extracts isolated from *Gynura japonica* at 100 mg/kg in mice.	Two oral doses of hyperoside at 20, 40, or 80 mg/kg on hours 6 and 30.	It dose-dependently reverses hepatic pathological damage, improves mitochondrial morphology, and enhances autophagy to achieve a protective effect.	[194]
Oral administration of senecionine at 50 mg/kg on day 5 in mice.	Oral administration of alisol B 23-acetate at 40 mg/kg on days 1–5.	It improves pathological liver damage and secondary electrolyte imbalances in the organism, which are related to the regulation of the AQP2 water transport pathway.	[205]
Single oral dose of senecionine at 50 mg/kg in mice.	Pretreatment (for 3 days) or post-treatment (on hours 6 and 30) of obeticholic acid (FXR agonist) at 20 mg/kg.	It reduces damage in the sinusoidal endothelium and abnormal serum markers, which involves improvement of FXR activity and excessive synthesis of downstream cholic acid species.	[182]
Treatment of clivorine at 50 μM for 48 h in L-02 cells.	Treatment of NAC at 5 mM for 24 h.	It decreases the DNA apoptotic ladder, expression of caspase-3 and caspase-9, and Cytc release. It rescues cellular GSH depletion.	[116]
Treatment of retrorsine at 50 μM for 24 h in primary rat hepatocytes.	Treatment of NAC at 2.5 mM for 24 h.	It alleviates oxidative stress, apoptosis, and autophagy.	[99]
Two oral doses of monocrotaline at 400 mg/kg/w for 1 week in mice.	Oral administration of sodium butyrate via drinking water at 200 mM for 1 week.	It delays PA-induced progression of HSOS, reduces mRNA expression of pro-inflammatory cytokines, which involves protection of the intestinal barrier, and reduces M1 liver macrophage polarization in the liver–gut axis.	[185]
Single oral dose of total alkaloid extracts of *Gynura japonica* at 100 mg/kg in mice.	Oral administration of chlorogenic acid at 20, 40, or 80 mg/kg for 2 times (at 6th and 30th hour) after total alkaloid extract exposure.	It reduces hepatic pathological changes and improves the imbalance of bile acid homeostasis, which involves activation of SIRT1/FXR pathway.	[196]

## Data Availability

No new data were created or analyzed in this study. Data sharing is not applicable to this article.

## Abbreviations

The following abbreviations are used in this manuscript:

AhR	Aryl hydrocarbon receptor
Abc	ATP-binding cassette subfamily
Akt	Protein kinase B
ALB	Albumin
ALP	Alkaline phosphatase
ALT	Alanine transaminase
ARE	Antioxidant response element
AST	Aspartate transaminase
ATF	Activating transcription factor
ATP	Adenosine triphosphate
Bak	Bcl-2 homologous antagonist killer
Bax	BCL2-associated X
BCL-2	B-cell lymphoma-2
Bcl-XL	B-cell lymphoma-extra large
BSO	Buthionine sulfonimine
CAT	Catalase
CHOP	C/EBP homologous protein
CYP	Cytochrome P450 enzymes
Cytc	Cytochrome c
DAMPs	Damage-associated molecular patterns
DB	Direct bilirubin
DHP	Dehydropyrrolizidine
Drp1	Dynamin-related protein 1
eIF-2α	Eukaryotic initiation factor-2α
ER	Endoplasmic reticulum
ERK	Extracellular signal-regulated kinase
FADD	Fas-associated death domain
Fas	Fas cell surface death receptor
FasL	Fas ligand
FMO	Flavin-containing monooxygenase
FoxO	Forkhead box protein O
FXR	Farnesoid X receptor
GCL	glutathione-cysteine lyase
GGT	Gamma-glutamyl transferase
GLCC	Glutamate–cysteine ligase catalytic subunit
GLCM	Glutamate–cysteine ligase modifier subunit
GPx	Glutathione peroxidase
GR	Glutathione reductase
GSH	Glutathione
GSSG	Glutathione oxidized
GST	Glutathione S-transferase
HILI	Herbal-induced liver injury
HSOS	Hepatic sinusoidal obstruction syndrome
IPA	Ingenuity Pathway Analysis
IRE1	Inositol-requiring enzyme 1
JNK	c-Jun N-terminal kinase
Keap1	Kelch-like ECH-associated protein 1
LC3	Microtubule-associated protein light chain 3
LDH	Lactate dehydrogenase
MAPK	Mitogen-activated protein kinase
MDA	Malondialdehyde
MDM2	Mouse double minute 2 homolog
MLKL	Mixed-lineage kinase domain-like protein
mTOR	Mammalian target of rapamycin
Myd88	Myeloid differentiation primary response protein 88
NAC	N-acetylcysteine
NADPH	Nicotinamide adenine dinucleotide phosphate
NF-κB	Nuclear factor kappa-B
NQO1	NAD(P)H dehydrogenase quinone 1
Nrf2	Nuclear factor erythroid 2-related factor 2
1-Oct	Organic cation transporter 1
p38	Mitogen-activated protein kinase 14
p53	Tumor protein 53
PAs	Pyrrolizidine alkaloids
PERK	Protein kinase R-like endoplasmic reticulum kinase
PSRC1	Proline/serine-rich coiled-coil 1
PXR	Pregnanediol X receptor
RID	Riddelliine
RIP	Receptor-interacting protein
RIPK3	Receptor-interacting serine/threonine kinase 3
ROS	Reactive oxygen species
RUCAM	Roussel Uclaf Causality Assessment Method
SAM	S-adenosyl methionine
SIRT	Sirtuin
Slc	Solute carrier family
Smad	Mothers against decapentaplegic homolog
SOD	Superoxide dismutase
TBA	Total bile acid
TBIL	Total bilirubin
TFEB	Transcription factor EB
TGF-β	Transforming growth factor-β
TLR	Toll-like receptors
TNF	Tumor necrosis factor
TP	Total protein
TRADD	TNFRSF1A-associated death domain
